# Gamblers' use of measures to prevent gambling problems and reduce harm

**DOI:** 10.3389/fpsyt.2022.857280

**Published:** 2022-07-27

**Authors:** Jonny Engebø, Torbjørn Torsheim, Ståle Pallesen

**Affiliations:** ^1^Department of Psychosocial Science, University of Bergen, Bergen, Norway; ^2^The Norwegian Gambling Authority, Førde, Norway; ^3^Norwegian Competence Centre for Gambling and Gaming Research, University of Bergen, Bergen, Norway; ^4^Optentia, The Vaal Triangle Campus of the North-West University, Vanderbijlpark, South Africa

**Keywords:** gambling problems, pre-commitment, prevention, harm reduction, responsible gambling, gambling, gamblers' protection

## Abstract

In this study, the use of measures to control gambling were investigated. Data from gamblers (*N* = 5,878) participating in a cross-sectional survey in 2019 based on random sampling from the Norwegian Population Registry, were analysed. The survey included questions about use of eight measures, which comprised the dependent variables. Questions about sociodemographics, gambling behaviour, gambling problems, self-reported impact from gambling advertisement and beliefs in measures to control gambling comprised the predictor variables. Logistic regression analyses were employed to identify significant predictors. Use of measures varied, ranging from 0.8% (contacting help services) to 23.2% (pre-commitment to affordable loss limits). All predictors had at least one significant association with the actual use of measures. Being a moderate risk or problem gambler was the most consistent predictor and was associated with the use of all eight measures. Being born outside Norway in a western or non-western country was associated with use of seven of the eight measures, whereas gambled online and participated in low-risk game only (inversely) were associated with use of six measures. Gender, age, game spending and beliefs in the usefulness of measures were associated with use of four measures. Participation in random games only was inversely associated with use of three measures. Self-reported impact from gambling advertisement was only (inversely) associated with self-testing for gambling problems. Several mechanisms responsible for the associations between predictors and the dependent variables are suggested, e.g., younger gamblers and moderate risk or problem gamblers may use these measures as they may acknowledge personal susceptibilities for developing gambling problems, such as impaired impulse control. Online gambling on the other hand was associated with use of various measures as the latter more often are integrated in online than offline gambling. Notably, the beliefs in measures as helpful was a significant predictor of use of four of the measures, which illustrates that positive views on the use of measures are not consistently associated with actual use of all the measures. Characteristics of the gamblers (e.g., place of birth, moderate risk or problem gambler), the game itself and the online distribution seem to be the most consistent predictors.

## Introduction

Gamblers have different measures they can use to control their own gambling and to reduce negative consequences. Most often such features are available at online gambling sites where gamblers e.g., can set restrictions on time and money spent on gambling. Among other measures, gamblers can also download an overview of their gambling expenditures. Such measures are often referred to as responsible gambling tools (RG) (1). In addition, gambling operators can have their own restrictions, e.g., maximum stake size, maximum loss limits and mandatory player breaks e.g., after 1 h continuously gambling. Further, gambling operators can monitor gambling behaviour and from that communicate with the gamblers. To receive help for gambling problems, gamblers can also contact help services as helplines, health personnel, other treatment providers and support groups. Some may also transfer control of their economy to others in order to prevent further problems or control gambling (2–4), typically implying a private agreement between the gambler and a trusted family member, a friend or a formally appointed guardian.

Several studies have investigated the use of tools to control gambling consumption. Most of them have investigated the use within online gambling environments and excluded land-based gambling. The emphasis on online gambling environments probably reflects that those tools often are based on registered play and hence more relevant for online gambling. One study, based on an online survey of 564 customers of Australian internet gambling sites found that among those who were aware of tools, most had accessed activity statements (88.4%), but fewer had used budget tools (24.5%) or taken timeouts (8.1%) (5). In another Australian study, account data of 39,853 gamblers wagering on sports and races were examined. In that study tools as deposit limits and timeouts are named Consumer Protection Tools (CPT). Most gamblers, 83%, did not use any CPT tools, deposit limits (15.8%), timeouts (0.55–1.57%) and self-exclusion (0.16–0.57%) (6).

In a study among Swedish gamblers who voluntary used the monitoring system Playscan, the gamblers could use several RG tools. In all, 26% had at that time used Playscan and of those 56% had set spending limits, 40% had taken a self-diagnostic problem gambling test and 17% had used a tool for self-exclusion (7).

A randomised control trial investigated the use of a deposit limit tool among gamblers on online slots who registered with a gambling company in Åland, (an autonomous island in Finland). For the gamblers who received a prompt/message about setting deposit limits at registration, before the first deposit or after the first deposit, the percentages of limit setters were 45.0, 38.8, and 21.9%, respectively. For the control group the percentage of limit setters was 6.5% (8).

Some studies have investigated the use of loyalty programs (9). Because loyalty schemes collect player data, such data can be used to prevent gambling problems. This could have been relevant for the land-based gambling e.g., in gambling arcades or casinos. A qualitative study among Finnish gamblers revealed mixed perceptions about a loyal customer program, which also offered gambling control tools. It was questioned if the program prevented gambling problems or actually increased consumption. The gambling control tools were regarded as useful but would not necessarily help problem gamblers (10). Studies in Australia have showed consistent positive associations between loyalty card use and risk gambling for venue-based gamblers (11).

Among those who suffer from gambling problems, few actually seek help (12, 13), and severe harm has often been experienced before contacting help (14).

This article addresses the question if gamblers use measures to control their gambling consumption. Knowledge of how gamblers use such measures or help is important for both gambling operators, help providers and regulators. Such knowledge for example helps to consider if some measures or features (e.g., budget tools) should be mandatory, and if help services should be more known and available for those who need help.

A Norwegian study with data from 2013, 2015 and 2019 showed that gamblers over the years have strengthened their beliefs in measures which can help them to control their gambling behaviour (15). In Norway, it is mandatory to set loss limits equal to or below maximum loss limits in some games. For many gamblers, these limits can still be set higher than what is affordable. In the present study, one of the variables measured if the set limits are low enough to be affordable.

A study analysing the data from 2013 and 2015 identified eleven variables as significant predictors of positive beliefs for the same measures: Female gender, young age, playing random games only, being a moderate risk or problem gambler, reporting high impact from gambling advertisements as well as the personality traits agreeableness, openness and neuroticism. Inversely, playing low risk games only, reporting a high amount of spending on gambling and the personality trait extraversion were related to less positive beliefs. Three variables showed no significant association with beliefs about RG measures: Place of birth (Norway or not), gambled online or not and the personality trait conscientiousness (3). Nine of the abovementioned variables were included in the present study. The five personality trait variables were not included.

This present study is the only one known to us which include a sample representative of the entire population of gamblers (i.e., participation in all types of available games, with both land-based and online distribution).

The present study has two research questions: (1) To what extent do the gamblers use measures to help them to control their gambling behaviour and (2) what can predict use of such measures when controlling for other relevant predictors / independent variables?

## Materials and methods

### Participants and sample

The present study is based on quantitative survey data stemming from a Norwegian prevalence study conducted by the University of Bergen (15). The data collection took place during the autumn of 2019. In total, 30,000 persons (gross sample) aged 16 through 74 years were randomly selected from the National Population Registry of Norway and invited to participate. Through a letter sent by postal mail, the invitation was first to respond to a web-based survey. With up to two reminders, it was also possible to participate by returning an enclosed paper-based questionnaire. In total 9,248 valid answers (net sample) were received. After eliminating persons with wrong addresses, illness, deaths, etc., an overall response rate of 32.7% was achieved. The response rate for similar postal surveys has been reduced since 2013 (43.6%) and 2015 (40.8%) (15). To reduce over- or under-representation among groups of gamblers, the data were weighted for age, gender and place of residence (county) in Norway.

In the weighted net sample, a total of 63.6% had gambled the last 12 months, 60.2% of the women (*n* = 4,742) and 67.1% of men (*n* = 4,506). Within specific age groups the gambling rate was lowest for those 16–25 years: 50.1% (*n* = 1,730). The other age groups had higher rates, 26–35 years: 64.1% (*n* = 1,806), 36–45 years: 66.7% (*n* = 1,644), 46–55 years: 68.2% (*n* = 1,628), 56–65 years: 67.4% (*n* = 1,401), and 66–74 years: 67.7% (*n* = 1,039). Among the gamblers 51.5% were male. In all, 0.7% of the gamblers were 16–17 years, 14.0% were 18–25, 73.3% were 26–65 years and 12.0% were 66–74 years, respectively. The mean age was 44.3 year, *SD* = 15.9 (*N* = 5,878).

### Procedure

The gamblers were categorised according to whether they had played low risk games only or if they had played games with higher risk (i.e., medium or high). Gamgard (an assessment tool) was used for this categorisation and divides games into very low, low, medium, high or very high risk, respectively. With this tool, ten game characteristics are considered with regards to a particular games' potential contribution to developing gambling problems, e.g., event frequency (time taken to buy a game, time from placing bet to the outcome, and time to buy the game again) and accessibility (how easily available a game is)^1^. The assessment tool also takes into consideration four RG features that moderate the risk, e.g., monetary budget tools^2^. These four RG features were not considered in the present assessment. In all 24.0% had played low risk games only (very low or low), whereas 76.0% had played at least one medium- or high-risk game (medium, high or very high). All the games are mentioned below (instrument section; games played and in **Table 3**). Number games, pools and a deposit bottle lottery were categorised as low risk games and all other games as higher risk (medium or high). As different games within one game category can have different risks, and since the questionnaire did not differentiate between all games within one category (e.g., for horse racing), the game type was consequently categorised as medium/high risk.

The gamblers were also categorised in terms of whether they had played at least one skill based game or random games only. Skill games imply games where the gamblers can improve their winner chances based on skills (i.e., pools, betting, horse racing, online poker and private games such as poker among friends). The non-skill or random games comprised number games, deposit bottle lottery, bingo and bingo machines, scratch cards, online casino, video lottery terminals (VLTs), and games on ships (slots and table games). Online casino and games on ships were categorised as random because the questions about these games did not differentiate between skill and non-skill games. A total of 64.5% of the gamblers had participated in random games only, whereas 35.5% had participated in at least one game involving skills.

To identify the gamblers who were most involved in at least one game type, the gamblers were also divided into two groups based on money spent. Those who had spent more than 5,000 NOK (~500 €) on at least one game type within the last 12 months were categorised as high spenders (comprising 11.1% of the gamblers), whereas those who had gambled for 5,000 NOK or less on every game (88.9% of the gamblers) were categorised as low spenders.

The gamblers were asked how often they gambled on four electronic devices: Stationary computer, lap-top, tablet or mobile phone. For each device, the response alternatives ranged from never to daily. In the present study an online gambler was defined as someone who had gambled online at least once during the 12 last months using at least one of the four devices. In total 58.4% were categorised as online gamblers, whereas 41.6% were categorised as land-based gamblers only.

### Instruments

#### Gambling participation

The respondents were asked if they during the last 12 months had participated in games (yes or no). The question contained a definition of games described as games with monetary stakes where results from an event or a draw could lead to monetary prizes.

#### Demographic

Because the sample was drawn from the National Population Registry, data on gender and age for each participant were provided from the registry. The respondents were asked about place of birth (eight alternatives: Norway, the other Nordic countries, the rest of Europe or one of the other five continents). Data on place of birth were used for making a dummy coded variable with three levels (born in Norway, born outside Norway either in Europe, North-America or Oceania (western countries), born in Africa, Asia, South or Central America (non-western countries). For the analyses, data for age was divided into four categories, 16–17, 18–25, 26–65, and 66–74 years, respectively.

#### Games played

The respondents were asked if they had participated in the following games: Number games, pools, betting, horse racing, bingo, bingo machines, scratch games, private games (e.g., poker games with friends), online casino, video lottery terminals (VLTs), games on ships (slots and table games), online poker and deposit bottle lottery. In addition to the Norwegian regulated games, the respondents were also asked if they had played games offered on foreign websites. The respondents confirmed participation by answering for each game the alternative for expenditure which was nearest to their gambling yearly spending (none/not gambled, NOK 1–1,000, NOK 1,001–5,000, NOK 5,001–10,000, NOK-10,001–25,000 and more than NOK 25,000) (1 NOK ~ 0.1 €). The questions were only answered by those who initially had confirmed that they had gambled the last 12 months. Those who had gambled were also asked if they had gambled online. From the collected data four dichotomous variables were constructed: Low risk games only vs. medium/high risk game participation, random games only vs. skill game participation, game spending (low vs. high) and online gambling (no vs. yes).

#### Canadian Problem Gambling Index (CPGI)

The CPGI was used to assess the extent of gambling problems. The CPGI consists of nine items related to gambling the last 12 months. Five of these items measure problematic gambling behaviour and four measures consequences (e.g., “Have you needed to gamble with larger amounts of money to get the same feeling of excitement?” and “Has gambling caused you any health problems, including stress or anxiety?”). The nine items are scored on a scale ranging from 0 (never) through 3 (always). The composite score thus varies from 0 to 27. Based on the composite score the respondents are divided into four groups: Non-problem gamblers (composite score 0), low risk gamblers (composite score 1 and 2), moderate risk gamblers (composite score 3 through 7) and problem gamblers (composite score 8 or higher) (16). In the analyses, the gamblers were divided into two groups: No problem/low risk gamblers and moderate risk/problem gambler. The prevalence of moderate risk or problem gamblers was 7.0% (*n* = 5,850). Cronbach's alpha for the CPGI in the present study was 0.91. Cronbach's alpha values above 0.70 are considered acceptable and values above 0.80 are preferable (17).

#### Impacts from gambling advertising

In all, nine items on how gambling advertising had an impact on the gamblers were included. Five of the items were adopted from the Effects of Gambling Advertising Questionnaire (EGAQ) (18). The items are scored from 1 (strongly disagree) through 4 (strongly agree). In addition, four items were added. Two of these were related to knowledge about gambling opportunities (“Gambling advertisement has increased my knowledge of gambling options” and “Gambling advertisement has increased my knowledge of gambling providers”). One item measured change in behaviour due to gambling advertisement (“I play with higher risk (use more money) because of gambling advertisements”) and one related to attitude (“I think more positively about gambling because of gambling advertisements”) (19). A total composite score was created by adding the score on each item divided by the number of items. These items were only answered by those who had gambled during the last 12 months. The mean total composite score was 2.02 (*SD* = 0.58, *N* = 5,764). Cronbach's alpha for the nine items was 0.82. For the analysis, the composite score was divided by median or nearest value into two groups, lower composite score and higher composite score.

#### Gamblers' belief in measures to control gambling behaviour

Ten items measured the gamblers' beliefs about measures in terms of how they think that these measures would help them to regulate their own gambling consumption. Many of the items were based on existing RG features, e.g., prize money direct to gamblers bank account and not directly available for further gambling (20). The questions were also based on an article that explored the perception of the value of potential RG measures (21). All the ten items covered mechanisms that are presently available in parts of the Norwegian gambling market (3). In the questionnaire, the gamblers were asked to which degree they agreed that these characteristics help or would help them regulating their own gambling consumption. There were five response alternatives for each item: Totally disagree = 1, disagree = 2, neither agree nor disagree = 3, agree = 4, and totally agree = 5. The mean total composite score was 3.37 (*SD* = 1.00, *N* = 5,771) and Cronbach's alpha was 0.95. For the analysis, the composite score was divided by median or nearest value into two groups, lower composite score and higher composite score.

#### Gamblers' use of measures to regulate their gambling behaviour

Six items measured if the gamblers had used external tools or features in the games to regulate their gambling, e.g., if the gambler had set amount limits in games low enough to not gamble more than one could afford or set a temporary brake in one or more games. One additional item measured if the gamblers had contacted help services for help because of one's own gambling problems and one assessed if the gambler had let others control his/her finances because of gambling problems. For each of the eight items the respondents could answer the following: “No”, “yes—during the last year” and “yes—but a longer time ago”. In the analyses, the two confirming categories were merged into one, thus the variables were dichotomized (never used the measure vs. used measures at least once). Table 1 shows the distribution or mean for the study variables.

**Table 1:** Percentages or mean and standard deviation (*SD*) of the studied variables among the gamblers (*N* = 5,677–5,878).

	**Percentage**	**Mean (** ***SD*** **)**
**Gender**		
Women	48.5	
Men	51.5	
Age (16–74)		44.27 (15.90)
16–17 years	0.7	
18–25 years	14.0	
26–65 years	73.3	
66–74 years	12.0	
**Place of birth**		
Europe, North America, Oceania	7.5	
Africa, Asia, South or Central America	3.4	
Norway	89.1	
**Participated in games with low or higher risk**		
Played higher risk games (medium or high)	76.0	
Played low risk games only	24.0	
**Participated in random or skill games**		
Played both random and skill games or skill only	35.5	
Played random games only	64.5	
**Game spending**		
Low	88.9	
High	11.1	
**Gambled online**		
No	41.6	
Yes	58.4	
**CPGI**		
Non-problem gambling (CPGI 0)	79.0	
Low-risk gambling (CPGI 1–2)	13.9	
Moderate risk gambling (3–7)	4.9	
Problem gambling (8+)	2.1	
Moderate risk of problem gamblers (CPGI 3+)	7.0	
**Impact from gambling advertisement**		2.02 (0.58)
Lower composite score—under median or nearest	49.5	
Higher composite score—over median or nearest	50.5	
**Beliefs about RG measures**		3.37 (1.00)
Lower composite score—under median or nearest	50.1	
Higher composite score—over median or nearest	49.9	
**Use of measures to control gambling^a^**		
Pre-committed to affordable amounts	23.2	
Set temporary player break(s) in one or more games	5.5	
Set a permanent exclusion in one or more games	2.8	
Self-tested to cheque for gambling problems	4.9	
Downloaded an overview of gambling expenses.	3.4	
Set a time-limit to restrict the gambling	3.4	
Contacted helpline, support groups or treatment	0.8	
Let others control the economy	1.0	

a Percentage who confirmed the use of such measures during the last year or earlier.

### Statistics

Results from all questions are presented in terms of frequencies or means with standard deviations. A cross-tabulation was conducted where the usage of measures was investigated separately against each independent variable. The results are presented in terms of percentages, chi-square values and phi or Cramer's V (effect sizes). Both the phi values and Cramer's V values indicate how strong effect the predictors have on the dependent variable. For the phi values, 0.1 is regarded as a low effect, 0.3 as a medium effect and 0.5 as a strong effect, respectively. For Cramer's V (with three degrees of freedom), 0.06 is regarded as a small effect, 0.17 as a medium effect and 0.29 as a strong effect, respectively (22).

The eight measures of gambling regulating behaviour comprised the dependent (dichotomized) variables. They were analysed separately with logistic regression analyses due to their substantial content specificity. Missing data was deleted pairwise. Independent variables comprised gender (women = 0, men = 1), age (16–17 year = 1, 18–25 year = 2, 26–65 year = 3 and 66–74 years = 4, where the latter comprised the contrast variable), dummy coding of place of birth (outside Norway in a western country = 1, Norway and non-western countries = 0; outside Norway in a non-western country = 1, Norway and other western countries = 0), game risk (middle/high = 0, low = 1), game type (at least one skill game = 0, random only = 1), game spending (low = 0, high = 1), online gambling (no = 0, yes = 1), being a moderate risk/problem gambler (no = 0, yes = 1), scores for self-reported impact from gambling advertisement (lower composite score = 0, higher composite score = 1) and scores for beliefs about RG measures (lower composite score = 0, higher composite score = 1). Preliminary analyses were conducted to ensure no violation of the assumption of multicollinearity. All variance inflation factors (VIF) had a value below 2.5. This is lower than 10.0 which is regarded as a threshold for problematic collinearity (23). Another and more conservative threshold suggestive of problematic collinearity is 2.5 (24).

## Results

Table 2 presents the eight items including the percentages endorsing and the corresponding 95% confidence intervals, it further shows that among the measures, the most used tool was being pre-committed to affordable amounts (23.2%). Fewer have confirmed the use of the other seven measures, which ranged from having set temporary break(s) in one or more games (5.5%) to having contacted helpline, support groups or treatment providers for help (0.8%).

**Table 2:** Percentage (including 95% confidence interval) for the eight items measuring self-regulation and help seeking for gambling problems (*N* = 5,733–5,761).

	**Percentage confirmed**	**95%** ***CI***	
		**Lower**	**Upper**
a. Pre-committed to affordable amounts	23.2	22.2	24.3
b. Set temporary player break(s) in one or more games	5.5	4.9	6.1
c. Set a permanent exclusion in one or more games	2.8	2.4	3.2
d. Taken a self-test to see if I might have a gambling problem	4.9	4.4	5.5
e. Downloaded an economical overview of my gambling	3.4	2.9	3.8
f. Set a time limit to restrict gambling longer than I have intended	3.4	2.9	3.9
g. Contacted helpline, support groups or treatment providers for help	0.8	0.6	1.0
h. Let others control my economy because of my gambling	1.0	0.8	1.3

Table 3 presents the prevalence of participation in different games or groups of games. The table also includes information about how games are distributed, and which games are grouped as random games and low risk games.

**Table 3:** Gamblers' participation in different games (*N* = 5,784–5,835).

**Games^a^**	**Distributed land-based (L) or online (O)**	**Random games**	**Low risk games**	**Participation percentage**
Number games (e.g., Lotto)	L and O	√	√	72.4
Scratch games, incl. from foreign websites	L and O	√		62.3
Bottle deposit lottery	L	√	√	38.5
Betting, incl. from foreign websites	L and O			18.4
Pools	L and O		√	14.1
Horseracing	L and O			10.9
Online casino games—incl. from foreign websites^b^	O	√		7.7
Private games (e.g., poker among friends)	L			7.7
Games on ships in international route traffic^b^	L (ships)	√		7.1
Online poker, offered from foreign websites only	O			5.5
Video lottery terminals (VLTs) in e.g., kiosks	L	√		4.0
Bingo games (main games) in bingo premises	L	√		3.7
Bingo machines (side games) in bingo premises	L	√		0.9
VLTs in bingo premises	L	√		1.0
Online bingo, incl. from foreign websites	O	√		3.0

a 4.6% (N = 5,745) answered also for an option other games. These games are not specified and not categorised. Therefore, not included.

b Most of the games offered are random (e.g., slots, roulette).

Table 4 presents the prevalence of problem gambling according to the four Canadian Problem Gambling Index categories, broken down by gender and age groups. The prevalence of problem gambling was highest for men, and for the younger age groups. The prevalence rate for those 16 to 17 years was the highest, however it should be noted that the sample size for this age group was small (*n* = 41), thus this estimate should be interpreted with caution.

**Table 4:** Percentage of gamblers in each Canadian Problem Gambling Index category, by gender and age.

	**Women**	**Men**	**16–17 years**	**18–25 years**	**26–65 years**	**66–74 years**
Normal gambler	84.6	73.8	58.5	62.1	80.8	89.0
Low risk gambler	11.2	16.4	14.6	24.8	12.8	8.3
Moderate risk gambler	2.8	6.9	14.6	10.3	4.3	1.7
Problem gambler	1.4	2.9	12.2	2.8	2.1	1.0
Total^a^^,^^b^	100.0	100.0	100.0	100.0	100.0	100.0
*n*	2,840	3,010	41	818	4,290	702
Moderate risk and problem gambler^c^^,^^d^	4.1	9.8	26.8	13.1	6.4	2.7

a For gender: Chi-Square (χ^2^) = 115.9, df = 3, p < 0.001.

b For age: Chi-Square (χ^2^) = 231.7, df = 9, p < 0.001.

c For gender: Chi-Square (χ^2^) = 70.4, df = 1, p < 0.001.

d For age: Chi-Square (χ^2^) = 93.0, df = 3, p < 0.001.

Table 5 shows percentages of gamblers in all groups who have used the different measures to prevent gambling problems. All predictors had at least four significant associations with the dependent variables. Highest phi (and strongest effect sizes; medium) was found for being a moderate risk or problem gambler setting temporary breaks in games (0.33) and letting others control their economy (0.31). Online gambling had a medium strong effect size for having pre-committed to affordable amounts (0.36). Highest Cramer's V (and strongest effect size; small) was found for age and having pre-committed to affordable amounts (0.12). Because of the significant associations found in the cross-table analyses, it was decided to include all independent variables (predictors) in logistic regression analyses.

**Table 5:** Gamblers who use the measures to prevent gambling problems. Percentage, chi-square (χ2) and phi (ϕ) and Cramer's V(ϕ_c_) *(N* = 5,572–5,762).

**Predictors**	**Pre-committed to affordable amounts**	**Set temporary player break(s)**	**Set a permanent exclusion**	**Taken a self-test for gambling problems**	**Downloaded an economical overview**	**Set a time limit—restricts gambling**	**Contacted help services for help**	**Let others control the economy**
Gender^a^	χ^2^ = 75.7^e^. ϕ = 0.115	χ^2^ = 45.1^e^. ϕ = 0.089	χ^2^ = 35.5^e^. ϕ = 0.080	χ^2^ = 61.3^e^. ϕ = 0.104	χ^2^ = 65.2^e^. ϕ = 0.107	χ^2^ = 17.3^e^. ϕ = 0.056	χ^2^ = 10.3^e^. ϕ = 0.044	χ^2^ = 17.1^e^. ϕ = 0.056
Female = 0	18.2%	3.4%	1.4%	2.6%	1.4%	2.4%	0.4%	0.4%
Male = 1	28.0%	7.5%	4.1%	7.1%	5.2%	4.4%	1.2%	1.6%
Age^b^	χ^2^ = 87.7^e^. ϕ_c_ = 0.123	χ^2^ = 47.7^e^. ϕ_c_ = 0.091	χ^2^ = 10.7^c^. ϕ_c_ = 0.043	χ^2^ = 30.1^e^. ϕ_c_ = 0.072	χ^2^ = 6.3^f^. ϕ_c_ = 0.033	χ^2^ = 49.0^e^. ϕ_c_ = 0.092	χ^2^ = 26.2^e^. ϕ_c_ = 0.067	χ^2^ = 39.1^e^. ϕ_c_ = 0.082
16–17 years	17.5%	15.4%	7.7%	5.1%	5.0%	7.7%	5.1%	10.3%
18–25 years	32.9%	9.9%	4.1%	6.4%	3.8%	7.1%	2.0%	1.5%
26–65 years	23.2%	5.1%	2.6%	5.3%	3.5%	3.0%	0.6%	0.9%
66–74 years	12.3%	2.8%	2.1%	0.7%	1.8%	1.0%	0.4%	0.3%
Country of birth^a^	χ^2^ = 5.3^c^. ϕ = 0.031	χ^2^ = 20.3^e^. ϕ = 0.061	χ^2^ = 18.6^e^. ϕ = 0.059	χ^2^ = 7.5^d^. ϕ = 0.038	χ^2^ = 6.8^d^. ϕ = 0.037	χ^2^ = 4.2^c^. ϕ = 0.029	χ^2^ = 8.4^d^. ϕ = 0.042	χ^2^ = 4.8^c^. ϕ = 0.032
Western. not Norway = 1	27.9%	10.5%	6.2%	7.9%	5.7%	5.3%	2.1%	2.1%
Non-western or Norway = 0	22.8%	5.1%	2.5%	4.7%	3.2%	3.2%	0.7%	0.9%
Country of birth^a^	χ^2^ = 9.2^d^. ϕ = 0.041	χ^2^ = 110.2^e^. ϕ = 0.141	χ^2^ = 36.8^e^. ϕ = 0.084	χ^2^ = 48.5^e^. ϕ = 0.0.95	χ^2^ = 39.1^e^. ϕ = 0.086	χ^2^ = 107.6^e^. ϕ = 0.140	χ^2^ = 100.0^e^. ϕ = 0.138	χ^2^ = 106.1^e^. ϕ = 0.141
Non-western. not Norway = 1	32.6%	23.1%	10.3%	16.1%	11.8%	17.2%	7.5%	8.6%
Western or Norway = 0	22.8%	4.9%	2.5%	4.6%	3.1%	2.9%	0.6%	0.7%
Low-risk games only^a^	χ^2^ = 232.8^e^ ϕ = −0.203	χ^2^ = 58.0^e^. ϕ = −0.102	χ^2^ = 24.4^e^. ϕ = −0.067	χ^2^ = 42.1^e^. ϕ = −0.087	χ^2^ = 39.7^e^. ϕ = −0.085	χ^2^ = 40.3^e^. ϕ = −0.085	χ^2^ = 5.1^c^. ϕ = −0.032	χ^2^ = 10.3^e^. ϕ = −0.045
No = 0 (only or also med/high risk)	28.2%	6.9%	3.4%	6.1%	4.3%	4.3%	1.0%	1.3%
Yes = 1	8.1%	1.4%	0.8%	1.6%	0.7%	0.7%	0.3%	0.2%
Participated in random games only^a^	χ^2^ = 297.1^e^. ϕ = −0.229	χ^2^ = 100.0^e^. ϕ = −0.133	χ^2^ = 62.9^e^. ϕ = −0.106	χ^2^ = 106.3^e^. ϕ = −0.137	χ^2^ = 163.6^e^. ϕ = −0.170	χ^2^ = 58.3^e^. ϕ = −0.102	χ^2^ = 26.8^e^. ϕ = −0.071	χ^2^ = 53.4^e^. ϕ = −0.099
No (only or also skill games) = 0	36.3%	9.7%	5.2%	9.0%	7.5%	5.9%	1.6%	2.3%
Yes = 1	16.1 %	3.3%	1.5%	2.7%	1.1%	2.0%	0.3%	0.3%
Game spending^a^	χ^2^ = 92.9^e^. ϕ = 0.130	χ^2^ = 113.6^e^. ϕ = 0.144	χ^2^ = 135.2^e^. ϕ = 0.157	χ^2^ = 45.0^e^. ϕ = 0.091	χ^2^ = 67.5^e^. ϕ = 0.111	χ^2^ = 28.3^e^. ϕ = 0.073	χ^2^ = 40.8^e^. ϕ = 0.089	χ^2^ = 112.9^e^. ϕ = 0.145
Low = 0	21.4%	4.4%	1.9%	4.4%	2.7%	3.0%	0.5%	0.5%
High = 1	38.8%	14.9%	10.1%	10.7%	9.1%	7.2%	3.0%	5.1%
Gambled online^a^	χ^2^ = 734.6^e^. ϕ = 0.358	χ^2^ = 127.9^e^. ϕ = 0.150	χ^2^ = 42.1^e^. ϕ = 0.087	χ^2^ = 138.8^e^. ϕ = 0.156	χ^2^ = 100.9^e^. ϕ = 0.134	χ^2^ = 79.9^e^. ϕ = 0.119	χ^2^ = 14.4^e^. ϕ = 0.052	χ^2^ = 20.5^e^. ϕ = 0.061
No = 0	5.3%	1.5%	1.1%	0.9%	0.5%	0.8%	0.3%	0.3%
Yes = 1	36.0%	8.4%	4.0%	7.8%	5.4%	5.2%	1.2%	1.5%
Moderate risk or problem gamblers^a^	χ^2^ = 182.8^e^. ϕ = 0.179	χ^2^ = 615.3^e^. ϕ = 0.329	χ^2^ = 427.5^e^. ϕ = 0.275	χ^2^ = 287.5^e^. ϕ = 0.225	χ^2^ = 202.5^e^. ϕ = 0.190	χ^2^ = 277.1^e^. ϕ = 0.222	χ^2^ = 290.6^e^. ϕ = 0.229	χ^2^ = 531.7^e^. ϕ = 0.308
No = 0	21.2%	3.5%	1.6%	3.6%	2.4%	2.3%	0.2%	0.2%
Yes = 1	51.0%	33.2%	19.4%	22.9%	15.9%	18.1%	8.3%	12.3%
Self-report. impact. gamb. adv.^a^	χ^2^ = 75.5^e^. ϕ = 0.116	χ^2^ = 36.4^e^. ϕ = 0.081	χ^2^ = 11.7^e^. ϕ = 0.047	χ^2^ = 9.6^d^. ϕ = 0.042	χ^2^ = 16.4^e^. ϕ = 0.055	χ^2^ = 35.0^e^. ϕ = 0.080	χ^2^ = 17.8^e^. ϕ = 0.058	χ^2^ = 27.6^e^. ϕ = 0.072
Lower composite score = 0	18.3%	3.6%	2.0%	4.0%	2.3%	1.9%	0.3%	0.3%
Higher composite score = 1	28.1%	7.3%	3.5%	5.8%	4.3%	4.8%	1.3%	1.6%
Beliefs in RG measures^a^	χ^2^ = 70.5^e^. ϕ = 0.112	χ^2^ = 9.3^d^. ϕ = 0.041	χ^2^ = 2.6^f^. ϕ = 0.023	χ^2^ = 4.7^c^. ϕ = 0.030	χ^2^ = 0.6^f^. ϕ = 0.004	χ^2^ = 16.2^e^. ϕ = 0.054	χ^2^ = 0.8^f^. ϕ = 0.014	χ^2^ = 1.4^f^. ϕ = 0.018
Lower composite score = 0	18.6%	4.6%	2.4%	4.3%	3.3%	2.5%	0.7%	0.8%
Higher composite score = 1	28.0%	6.5%	3.2%	5.6%	3.5%	4.4%	0.9%	1.2%

a df = 1.

b df = 3 and Cramer's V (ϕ_c_).

c p < 05,

d p < 0.01,

e p < 0.001,

f Non-significant.

The results from the regression analysis are shown in Table 6. Measured by Nagelkerke R Square, the eleven predictors in total explained between 19.6% (setting a time limit which restricts the gambling) and 40.9% (letting others control the gamblers economy) of the variance.

**Table 6:** Logistic regression analyses. Summary for predicting the use of eight different measures to control gambling *(N* = 5,365–5,377).

**Predictors**	**Pre-committed to affordable amounts**		**Set temporary player break(s)**		**Set a permanent exclusion**		**Taken a self-test for gambling problem**		**Downloaded an economical overview**		**Set a time limit which restricts the gambling**		**Contacted help services for help**		**Let others control the economy**	
	***OR***	**95%** ***CI***^a^	***OR***	**95%** *CI*^a^	***OR***	**95%** *CI*^a^	***OR***	**95%** *CI*^a^	***OR***	**95%** *CI*^a^	***OR***	**95%** *CI*^a^	***OR***	**95%** *CI*^a^	***OR***	**95%** *CI*^a^
Gender (female = 0. male = 1)	1.10	(0.94–1.29)	1.38	(1.03–1.86)	1.69	(1.11–2.58)	1.75	(1.29–2.37)	1.89	(1.26–2.81)	1.09	(0.77–1.55)	1.19	(0.54–2.61)	1.24	(0.58–2.62)
Age (16–17)^c^	0.81	(0.29–2.21)	2.64	(0.74–9.51)	1.41	(0.31–6.35)	2.38	(0.38–14.84)	0.82	(0.14–4.89)	2.91	(0.58–14.50)	3.30	(0.23–47.83)	n.a.^b^	n.a.
Age (18–25)^c^	1.86	(1.32–2.61)	2.30	(1.17–4.50)	1.32	(0.59–2.97)	4.38	(1.47–13.06)	0.87	(0.39–1.95)	3.72	(1.40–9.86)	4.53	(0.60–34.26)	n.a.^b^	n.a.
Age (26–65)^c^	1.24	(0.92–1.67)	1.29	(0.69–2.41)	0.93	(0.46–1.91)	5.24	(1.83–14.95)	1.13	(0.55–2.31)	1.87	(0.73–4.79)	1.36	(0.19–9.70)	n.a.^b^	n.a.
Born outside Norway in a western country (no = 0. yes = 1)	1.69	(1.29–2.21)	2.43	(1.64–3.60)	2.62	(1.59–4.32)	1.66	(1.09–2.52)	1.92	(1.17–3.14)	1.83	(1.10–3.04)	4.25	(1.76–10.29)	1.83	(0.78–4.31)
Born outside Norway in a non-western country (no = 0. yes = 1)	1.40	(0.96–2.05)	4.00	(2.53–6.34)	2.47	(1.33–4.59)	2.82	(1.73–4.58)	3.50	(2.01–6.09)	4.67	(2.86–7.62)	8.28	(3.67–18.70)	4.08	(1.84–9.04)
Participated in low-risk games only (no = 0. yes = 1)	0.29	(0.23–0.36)	0.36	(0.21–0.60)	0.47	(0.24–0.94)	0.46	(0.28–0.74)	0.42	(0.20–0.86)	0.32	(0.16–0.65)	1.74	(0.53–5.73)	1.69	(0.45–6.27)
Participated in random games only (no = 0. yes = 1)	0.85	(0.72–1.00)	0.99	(0.73–1.34)	0.89	(0.58–1.37)	0.77	(0.57–1.04)	0.35	(0.23–0.53)	0.80	(0.56–1.16)	0.52	(0.21–1.25)	0.34	(0.14–0.83)
Game spending (low = 0. high = 1)	1.68	(1.36–2.09)	2.04	(1.47–2.84)	2.96	(1.97–4.44)	1.19	(0.84–1.68)	1.35	(0.92–1.98)	1.28	(0.84–1.97)	2.04	(0.96–4.34)	2.60	(1.34–5.07)
Gambled online (no = 0. yes = 1)	9.68	(7.77–12.05)	4.87	(3.16–7.49)	2.35	(1.39–3.96)	6.14	(3.79–9.94)	6.81	(3.42–13.55)	4.13	(2.48–6.88)	1.99	(0.67–5.91)	1.37	(0.47–3.96)
Moderate risk or problem gamblers (no = 0. yes = 1)	1.44	(1.12–1.85)	4.98	(3.65–6.80)	5.70	(3.74–8.69)	3.64	(2.60–5.10)	2.69	(1.81–4.01)	3.10	(2.10–4.57)	8.77	(3.95–19.48)	19.86	(8.56–46.09)
Self-report. impact. gamb. adv. Lower composite score = 0 Higher composite score = 1	1.08	(0.93–1.25)	1.02	(0.77–1.34)	0.89	(0.60–1.31)	0.74	(0.57–0.98)	1.07	(0.76–1.49)	1.24	(0.88–1.75)	1.75	(0.73–4.19)	1.82	(0.77–4.33)
Beliefs in RG measures Lower composite score = 0 Higher composite score = 1	1.76	(1.52–2.04)	1.39	(1.07–1.81)	1.23	(0.86–1.77)	1.32	(1.01–1.72)	1.04	(0.76–1.43)	1.61	(1.16–2.23)	1.06	(0.55–2.05)	1.15	(0.62–2.16)
Cox and Snell R Square	0.203		0.091		0.051		0.066		0.057		0.051		0.029		0.042	
Nagelkerke R Square	0.307		0.262		0.227		0.198		0.222		0.196		0.322		0.409	

a Significant relationships, p ≤ 0.05, are shown in bold.

b Results not available due to small number of observations.

c Reference group is age group 66–74.

For all, but one dependent variable, there were several significant predictors. Male gender was associated with increased probabilities of using four of the measures (set temporary player breaks, set a permanent exclusion, taken a self-test for gambling problem and downloaded an economical overview). Compared to the contrast group (age 66–74), younger age (18–25 years) was related to higher probability of using four of the measures (pre-commitment to affordable amounts, set temporary player breaks, taken a self-test for gambling problems and set a time limit which restricts gambling). Also, the age group 26–65 had a higher probability to take a self-test for gambling problems. Being born outside Norway, in another western or in a non-western country was associated with increased probability of having used in total all the eight measures. Having gambled with low risk games only was associated with lower probability of having used six of the eight measures (pre-commitment to affordable amounts, set temporary player breaks, set a permanent exclusion, taken a self-test for gambling problem, downloaded an economical overview and set a time limit which restricts gambling). Having participated in random games only was associated with lower probabilities of having used three of the eight measures (pre-committed to affordable amounts, downloaded an economical overview and let others control the economy).

Being a high spender was associated with increased probability of having used four of the eight measures (pre-commitment to affordable amounts, set temporary player breaks, set a permanent exclusion and let others control the economy). Having gambled online was associated with increased probability of having used six of the eight measures (pre-commitment to affordable amounts, set temporary player breaks, set a permanent exclusion, taken a self-test for gambling problem, downloaded an economical overview and set a time limit which restricts gambling). Being a moderate risk gambler or a problem gambler was associated with an increased probability of having used all the eight measures (pre-committed to affordable amounts, set temporary player breaks, set a permanent exclusion, taken a self-test for gambling problem, downloaded an economical overview, set a time limit which restricts gambling, contacted help services for help and let others control the economy). Self-reported impact from gambling advertisements was only associated with a decreased probability of having taken a self-test for gambling problems. Finally, beliefs in real or potential help from RG-measures was associated with an increased probability of having used four of the eight measures (pre-commitment to affordable amounts, set temporary player breaks, taken a self-test for gambling problem, and set a time limit which restricts gambling).

The dependent variable associated with the fewest (three) predictors was contacting help services, whereas setting temporary player break and taken a self-test were the dependent variables associated with most (nine) predictors. All predictors showed significant relationships to at least one dependent variable.

## Discussion

Generally, the measures in question were used by a relatively small proportion of the gamblers. This is in line with other studies, reporting that only a minority of gamblers actively use tools to regulate their gambling behaviours (e.g., to set money limits) (25). The most used measure was to set limits for affordable amounts.

All the predictors showed significant associations with the use of measures which can control or reduce harm from gambling. The significant associations for the different predictors ranged from one for self-reported impact from gambling advertisement to eight for being a moderate risk or problem gambler.

Male gamblers reported to have used four of the measures (set temporary breaks, set a permanent exclusion, taken a self-test of gambling problems and downloaded an economical overview of gambling expenditures) more often than female gamblers. Although we previously have shown that women have more positive beliefs in the effect of external measures to control gambling (3), the present result shows that males used several of them more frequently. An explanation to this might be that men in general are more strongly involved in gambling. In line with this a Swedish study showed female preponderance in a group of “seldom gamblers” whereas the gender distribution was almost equal in the group of occasional gamblers. For the groups of habitual, social and heavy gamblers, the vast majorities were males (26). A Finnish study of people aged 18–29 showed that frequent gambling, playing several game types, online gambling and at risk-or problem gambling occurred more often among males than females (27). Because of the larger gambling involvement and the fact that men generally take more risks the women (28), a suggested explanation for the gender association with measures to control gambling is that men more often than women need and therefore also actually use these features or measures to control their gambling more frequently.

Younger age (18–25 years) was a significant predictor for the actual use of four measures (pre-commitment to setting loss limits, setting temporary breaks, taking a self-test for gambling problems and setting a time limit to restrict gambling). In addition, those 26–65 years self-tested more often than the reference group (66–74 years). This is in line with the aforementioned study (3) which showed that younger gamblers had stronger beliefs in measures to control gambling than older gamblers. Younger subjects have generally been found to take more risks than older subjects (29). Young age is also a risk factor for problem gambling (30). Based on this, it is conceivable that younger gamblers more often than older gambler see measures as useful to control their gambling.

Being born outside Norway, both in western and non-western countries were each significant predictors of seven of the measures, with the exception of pre-commitment to setting loss-limits (for being born outside Norway in a non-western country) and letting others control the economy (for being born outside Norway in a western country), as gamblers not born in Norway used the measures more often. This is in line with an Australian study where gamblers who spoke a language other than English had used a significantly greater number of consumer protection tools (5). In our data, there are no obvious and direct explanations to the findings. However, the findings may reflect cultural factors such as acculturation processes, cultural beliefs about gambling and stigmas associated with gambling behaviour and gambling problems (31–33), religious factors, such as participation in religious rituals (34) as well as financial factors such as lower income among immigrants than native born^3^. Further explanations can be that those born outside Norway to a larger extent than natives are exposed to gambling opportunities (e.g., available time, service occupations) (35), rendering them in a greater need of regulatory measures.

Gambling on low-risk games only was inversely associated with the use of six measures (pre-commitment to setting loss limits, setting temporary breaks, setting a permanent exclusion, taking a self-test for gambling problems, downloading of an economical overview of gambling expenditures and setting a time limit to restrict gambling). One explanation for this finding is that those gambling on low-risk games only, keep control and seldom need these external measures as there is a natural restriction inherent in the games themselves (36, 37). In accordance with this we have previously showed that those gambling on low-risk games only also more seldom have beliefs that measures will help them to control their gambling (3).

Participation in random games only was inversely associated with the use of three measures (pre-committed to affordable amounts, downloading of an economical overview of gambling expenditures and letting other control the economy). Hence, skill game gamblers used the three aforementioned measures more often than those who participated in random games only. Participation in skill games has been linked with “illusion of control” (30), where gamblers may be over-confident about their own skills when gambling. Illusion of control has further been associated with gambling persistence (38). Hence, those gambling non-random games may over time develop more problems, which eventually may force them to employ measures to control their gambling behaviour. In this study we specifically found that gamblers of skill games more often than others pre-committed to affordable amounts, downloaded an overview of gambling expenses and let others control their economy.

The gamblers with high spending were more likely to use four of the measures (pre-commitment to setting loss limits, setting temporary breaks, setting a permanent exclusion, and letting other control their economy). The same group of gamblers reported however fewer positive beliefs in such measures than low spenders (3). A suggested explanation in that study is that measures would restrict their gambling and it could thus be assumed that measures to control gambling behaviours therefore would be less welcomed by these gamblers. However, the result of the present study paints a different picture and suggests that when the spending become sufficiently high the actual use of measures to control gambling is deemed as a necessary evil among high spending gamblers.

Gamblers who had gambled online had significant higher use of six measures (pre-commitment to setting loss limits, setting temporary breaks, setting a permanent exclusion, taking a self-test for gambling problems, downloading of an economical overview of gambling expenditures and setting a time limit to restrict gambling). These findings are expected as online gambling to a greater extent than land-based gambling enables enforcement of different measures to control and regulate gambling behaviour (2).

Moderate risk or problem gamblers used all the eight measures (pre-committed to affordable amounts, setting temporary breaks, setting a permanent exclusion, taking a self-test for gambling problems, downloading of an economical overview of gambling expenditures, setting a time limit to restrict gambling, contacting/seeking help and letting others control the economy) more frequently than those with milder or no problems. These findings are also expected since the former group has a stronger need for external measures to restrict/control their gambling (39).

In the study by Engebø et al., the gamblers who reported stronger impact from gambling advertisement assessed external measures as more helpful or potentially more helpful to control gambling than those reporting less impact from gambling advertisement (3). When it comes to actual use, the present study showed that experienced impact from gambling advertisement was associated with the use of one measure only—a decreased use of self-tests for gambling problems.

The last predictor investigated was the composite score for beliefs in RG measures. Actual use of four measures (pre-commitment to loss limits, setting temporary breaks, taking a self-test for gambling problems and setting a time limit to restrict gambling) had a higher probability when the belief was stronger for such measures. This illustrates that there for several measures is a positive association between beliefs in the usefulness of such measures and actual use.

### Practical implications

Different groups of gamblers can have different views on measures to control their gambling and different characteristics of the gamblers as well as of the games may be related to actual use of the measures. The present study suggests that belief in the usefulness of RG measures is associated with actual use of such measures, although not consistently. Future research should accordingly identify factors that can in more detail explain the relationship between views of and actual use of external measures. The present study shows that moderate risk or problem gamblers use all the measures more often. Also, gamblers borns outside Norway use the measures more often than native born, although obvious explanations for this are not available or known. Knowledge about predictors of use and the views of external measures are important for operators and regulators as such knowledge may pinpoint who underuses such measures and who seems to need them the most. The results are also relevant for the discourse concerning whether or not measures to control or regulate gambling behaviour should be voluntarily or mandatory (25).

### Strengths and limitations

To the best of our knowledge the present study is the only known to us which is based on a representative sample of the entire population of gamblers (i.e., participation in all types of available games, with both land-based and online distribution) investigating actual use of measures to control gambling or reduce consequences. Although the sample size was relatively large and sufficient for all the main analysis, it was too small to justify analyses broken down by individual games.

The present paper comprises gamblers' self-reported use of measures. This should be of interest to e.g., gambling operators and regulators. However, the analysis would have been more precise and actual if the data were based on actual use from registered play. It is also a limitation that some of the data are too non-specific for analysis. Data on place of birth was for example limited to continents and not countries. Since e.g., cultures might differ across a continent, specific cultural explanations to the findings were difficult to obtain based on the present data. Another limitation is the small group of the youngest gamblers (16–17 years). The data for the present study stems from a prevalence study in the adult population in Norway. The age interval for Norwegian gambling problem prevalence studies has been 16–74 years since 2007 (15). Gambling among youths is an important issue and should be investigated in larger youth studies where sample sizes are more sufficient for this age group. The final limitation concerns the relatively low response rate (32.7%) implying that the majority of those invited did not reply. Over- and under-representation in the data were compensated for by weighting the sample for gender, age and place of residence (county). However, we cannot rule out that those who did not participate also differed from those who did on other parameters. Previous research has for example shown that those who participate in surveys generally are more resourceful and with better health than those who do not participate (40). Thus, it is possible that this has limited the generalizability of the findings.

### Conclusions

Gamblers, to varying degree use external measures to control their gambling behaviour. The most often used measure was to pre-commit to loss limits. Six of the measures were mostly available as features from online gambling websites. These measures can be used to prevent gambling problems, but also to reduce negative consequences from problematic gambling. Two other measures are activities which are more relevant when excessive gambling has reached a certain level (i.e., contacting help or treatment services and letting others control the economy).

All predictors had at least one significant association with the actual use of measures. Only one and a negative association was found for self-reported impact from gambling advertisement (self-test for gambling problems). Being a moderate risk or problem gambler or being born outside Norway were the most consistent predictors, being associated with, respectively, eight and seven of the eight measures. Overall, both characteristics of the gambler (e.g., male gender, young age and reporting gambling problems) and characteristics of the games (e.g., skill, online) were associated with the use of measures to regulate gambling behaviour. Although gamblers' belief in measures as helpful was a significant predictor of four of the measures, other predictors showed a more consistent relationship with the measures. This illustrates that positive views of the measures to some extents are associated with actual behaviour.

## Data availability statement

The data analyzed in this study is subject to the following licenses/restrictions: Data are available on request. Requests to access the dataset should be directed to JE, jonny.engebo@lottstift.no.

## Ethics statement

All procedures used in the present study were conducted in accordance with the 1964 Helsinki Declaration and its later amendments. The present study was approved by the Norwegian Centre for Research Data (no. 528056).

## Author contributions

JE has conducted the analysis and drafted the first version of the manuscript. All authors contributed to the interpretation of data, revised the work critically for important intellectual content, approved the final version to be published, and agreed to be accountable for all aspects of the work in ensuring that questions related to the accuracy or integrity of any part of the work are appropriately investigated and resolved.

## Conflict of interest

JE works as a senior adviser with The Norwegian Gambling Authority where one of his major tasks is related to regulation and responsible gambling. In addition, JE is a board member of GREF (Gaming Regulators European Forum) and he is also co-chair of a GREF working group in responsible gambling. Further he is a member of the executive committee of EASG (The European Association for the Study of Gambling). The remaining authors declare that the research was conducted in the absence of any commercial or financial relationships that could be construed as a potential conflict of interest.

## Publisher's note

All claims expressed in this article are solely those of the authors and do not necessarily represent those of their affiliated organizations, or those of the publisher, the editors and the reviewers. Any product that may be evaluated in this article, or claim that may be made by its manufacturer, is not guaranteed or endorsed by the publisher.

## Footnotes

^1^Gamgard, www.gamgard.com (Accessed April 27, 2022).

^2^Gamres, www.gamres.org (Accessed April 27, 2022).

^3^Statistics Norway. (2021). SSB analyse 2021/06 Utdanning og lønnsnivå hos innvandrere, [Education and income level for immigrants]. Retrieved from https://www.ssb.no/arbeid-og-lonn/artikler-og-publikasjoner/utdanning-og-lonnsniva-hos-innvandrere (Accessed April 27, 2022).
